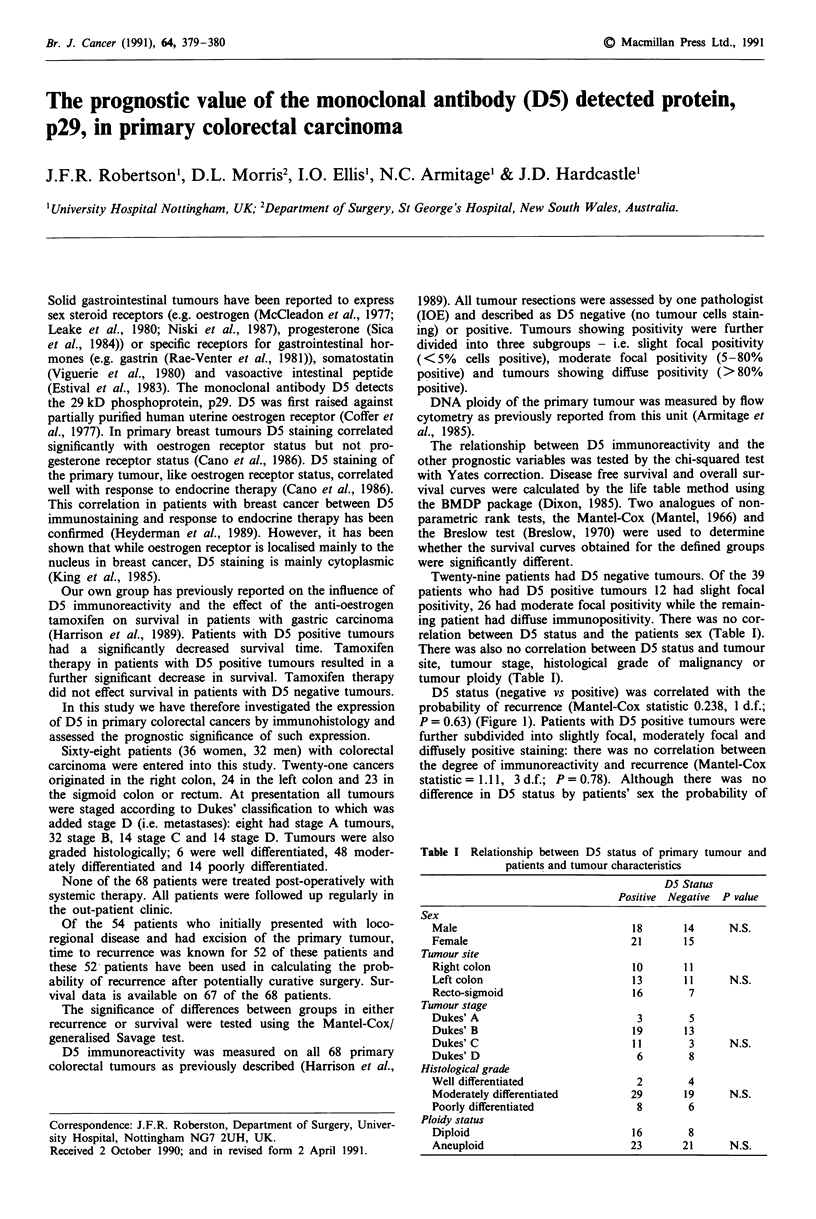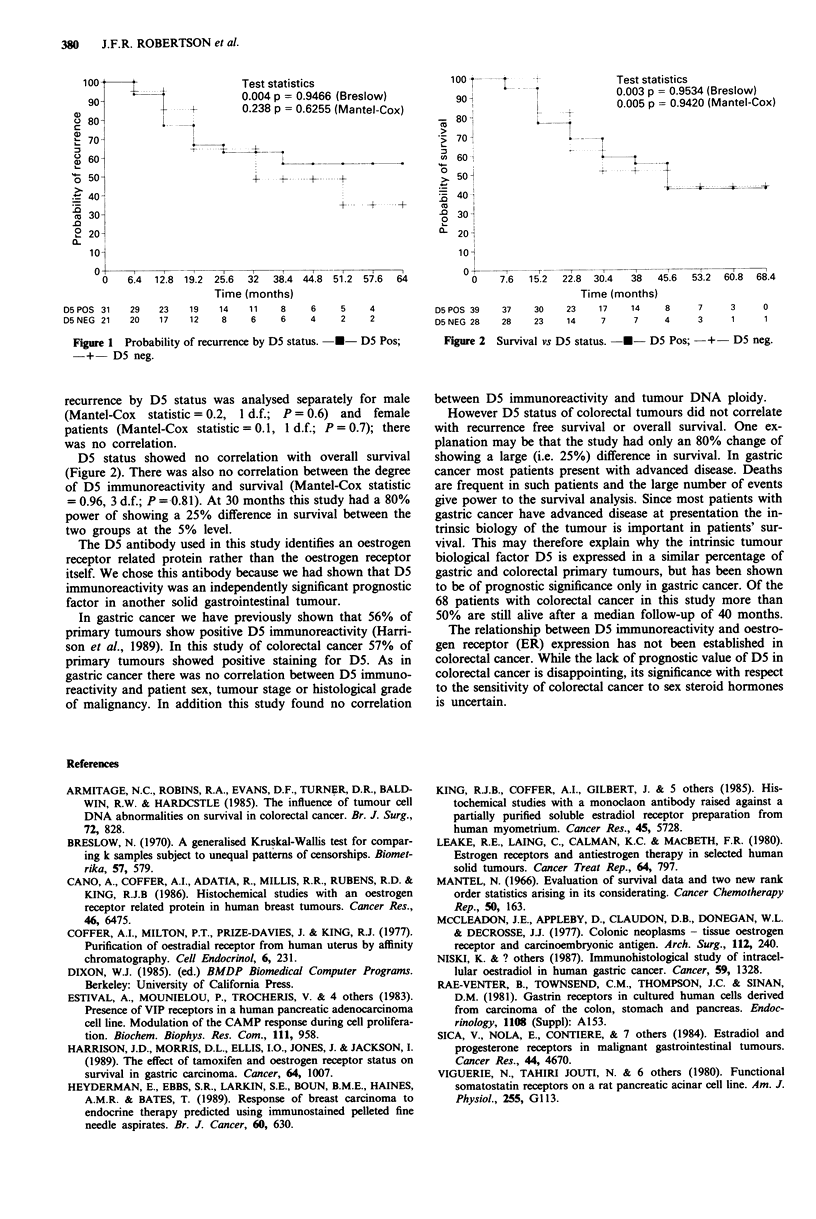# The prognostic value of the monoclonal antibody (D5) detected protein, p29, in primary colorectal carcinoma.

**DOI:** 10.1038/bjc.1991.312

**Published:** 1991-08

**Authors:** J. F. Robertson, D. L. Morris, I. O. Ellis, N. C. Armitage, J. D. Hardcastle

**Affiliations:** University Hospital Nottingham, UK.


### The prognostic value of the monoclonal antibody (D5) detected protein, p29, in primary colorectal carcinoma

J.F.R. Robertson[1], D.L. Morris[2], I.O. Ellis[1], N.C. Armitage[1] & J.D. Hardcastle[1]

[1]*University Hospital Nottingham, UK;* [2]*Department of Surgery, St George's Hospital, New South Wales, Australia.*

Solid gastrointestinal tumours have been reported to express sex steroid receptors (e.g. oestrogen (McCleadon *et al.*, 1977; Leake *et al.*, 1980; Niski *et al.*, 1987), progesterone (Sica *et al.*, 1984)) or specific receptors for gastrointestinal hormones (e.g. gastrin (Rae-Venter *et al.*, 1981)), somatostatin (Viguerie *et al.*, 1980) and vasoactive intestinal peptide (Estival *et al.*, 1983). The monoclonal antibody D5 detects the 29 kD phosphoprotein, p29. D5 was first raised against partially purified human uterine oestrogen receptor (Coffer *et al.*, 1977). In primary breast tumours D5 staining correlated significantly with oestrogen receptor status but not progesterone receptor status (Cano *et al.*, 1986). D5 staining of the primary tumour, like oestrogen receptor status, correlated well with response to endocrine therapy (Cano *et al.*, 1986). This correlation in patients with breast cancer between D5 immunostaining and response to endocrine therapy has been confirmed (Heyderman *et al.*, 1989). However, it has been shown that while oestrogen receptor is localised mainly to the nucleus in breast cancer, D5 staining is mainly cytoplasmic (King *et al.*, 1985).

Our own group has previously reported on the influence of D5 immunoreactivity and the effect of the anti-oestrogen tamoxifen on survival in patients with gastric carcinoma (Harrison *et al.*, 1989). Patients with D5 positive tumours had a significantly decreased survival time. Tamoxifen therapy in patients with D5 positive tumours resulted in a further significant decrease in survival. Tamoxifen therapy did not effect survival in patients with D5 negative tumours.

In this study we have therefore investigated the expression of D5 in primary colorectal cancers by immunohistology and assessed the prognostic significance of such expression.

Sixty-eight patients (36 women, 32 men) with colorectal carcinoma were entered into this study. Twenty-one cancers originated in the right colon, 24 in the left colon and 23 in the sigmoid colon or rectum. At presentation all tumours were staged according to Dukes' classification to which was added stage D (i.e. metastases): eight had stage A tumours, 32 stage B, 14 stage C and 14 stage D. Tumours were also graded histologically; 6 were well differentiated, 48 moderately differentiated and 14 poorly differentiated.

None of the 68 patients were treated post-operatively with systemic therapy. All patients were followed up regularly in the out-patient clinic.

Of the 54 patients who initially presented with loco-regional disease and had excision of the primary tumour, time to recurrence was known for 52 of these patients and these 52 patients have been used in calculating the probability of recurrence after potentially curative surgery. Survival data is available on 67 of the 68 patients.

The significance of differences between groups in either recurrence or survival were tested using the Mantel-Cox/generalised Savage test.

D5 immunoreactivity was measured on all 68 primary colorectal tumours as previously described (Harrison *et al.*, 1989). All tumour resections were assessed by one pathologist (IOE) and described as D5 negative (no tumour cells staining) or positive. Tumours showing positivity were further divided into three subgroups – i.e. slight focal positivity (<5% cells positive), moderate focal positivity (5–80% positive) and tumours showing diffuse positivity (>80% positive).

DNA ploidy of the primary tumour was measured by flow cytometry as previously reported from this unit (Armitage *et al.*, 1985).

The relationship between D5 immunoreactivity and the other prognostic variables was tested by the chi-squared test with Yates correction. Disease free survival and overall survival curves were calculated by the life table method using the BMDP package (Dixon, 1985). Two analogues of non-parametric rank tests, the Mantel-Cox (Mantel, 1966) and the Breslow test (Breslow, 1970) were used to determine whether the survival curves obtained for the defined groups were significantly different.

Twenty-nine patients had D5 negative tumours. Of the 39 patients who had D5 positive tumours 12 had slight focal positivity, 26 had moderate focal positivity while the remaining patient had diffuse immunopositivity. There was no correlation between D5 status and the patients sex (Table I). There was also no correlation between D5 status and tumour site, tumour stage, histological grade of malignancy or tumour ploidy (Table I).

D5 status (negative *vs* positive) was correlated with the probability of recurrence (Mantel-Cox statistic 0.238, 1 d.f.; $P = 0.63$) (Figure 1). Patients with D5 positive tumours were further subdivided into slightly focal, moderately focal and diffusely positive staining: there was no correlation between the degree of immunoreactivity and recurrence (Mantel-Cox statistic = 1.11, 3 d.f.; $P = 0.78$). Although there was no difference in D5 status by patients' sex the probability of

**Table I** Relationship between D5 status of primary tumour and patients and tumour characteristics

| | *D5 Status* | | |
|---|---|---|---|
| | *Positive* | *Negative* | *P value* |
| *Sex* | | | |
| Male | 18 | 14 | N.S. |
| Female | 21 | 15 | |
| *Tumour site* | | | |
| Right colon | 10 | 11 | |
| Left colon | 13 | 11 | N.S. |
| Recto-sigmoid | 16 | 7 | |
| *Tumour stage* | | | |
| Dukes' A | 3 | 5 | |
| Dukes' B | 19 | 13 | |
| Dukes' C | 11 | 3 | N.S. |
| Dukes' D | 6 | 8 | |
| *Histological grade* | | | |
| Well differentiated | 2 | 4 | |
| Moderately differentiated | 29 | 19 | N.S. |
| Poorly differentiated | 8 | 6 | |
| *Ploidy status* | | | |
| Diploid | 16 | 8 | |
| Aneuploid | 23 | 21 | N.S. |

Correspondence: J.F.R. Roberston, Department of Surgery, University Hospital, Nottingham NG7 2UH, UK.
Received 2 October 1990; and in revised form 2 April 1991.

**Figure 1** Probability of recurrence by D5 status. —■— D5 Pos; —+— D5 neg.

**Figure 2** Survival *vs* D5 status. —■— D5 Pos; —+— D5 neg.

recurrence by D5 status was analysed separately for male (Mantel-Cox statistic = 0.2, 1 d.f.; $P = 0.6$) and female patients (Mantel-Cox statistic = 0.1, 1 d.f.; $P = 0.7$); there was no correlation.

D5 status showed no correlation with overall survival (Figure 2). There was also no correlation between the degree of D5 immunoreactivity and survival (Mantel-Cox statistic = 0.96, 3 d.f.; $P = 0.81$). At 30 months this study had a 80% power of showing a 25% difference in survival between the two groups at the 5% level.

The D5 antibody used in this study identifies an oestrogen receptor related protein rather than the oestrogen receptor itself. We chose this antibody because we had shown that D5 immunoreactivity was an independently significant prognostic factor in another solid gastrointestinal tumour.

In gastric cancer we have previously shown that 56% of primary tumours show positive D5 immunoreactivity (Harrison *et al.*, 1989). In this study of colorectal cancer 57% of primary tumours showed positive staining for D5. As in gastric cancer there was no correlation between D5 immunoreactivity and patient sex, tumour stage or histological grade of malignancy. In addition this study found no correlation between D5 immunoreactivity and tumour DNA ploidy.

However D5 status of colorectal tumours did not correlate with recurrence free survival or overall survival. One explanation may be that the study had only an 80% change of showing a large (i.e. 25%) difference in survival. In gastric cancer most patients present with advanced disease. Deaths are frequent in such patients and the large number of events give power to the survival analysis. Since most patients with gastric cancer have advanced disease at presentation the intrinsic biology of the tumour is important in patients' survival. This may therefore explain why the intrinsic tumour biological factor D5 is expressed in a similar percentage of gastric and colorectal primary tumours, but has been shown to be of prognostic significance only in gastric cancer. Of the 68 patients with colorectal cancer in this study more than 50% are still alive after a median follow-up of 40 months.

The relationship between D5 immunoreactivity and oestrogen receptor (ER) expression has not been established in colorectal cancer. While the lack of prognostic value of D5 in colorectal cancer is disappointing, its significance with respect to the sensitivity of colorectal cancer to sex steroid hormones is uncertain.

### References

ARMITAGE, N.C., ROBINS, R.A., EVANS, D.F., TURNER, D.R., BALDWIN, R.W. & HARDCSTLE (1985). The influence of tumour cell DNA abnormalities on survival in colorectal cancer. *Br. J. Surg.*, **72,** 828.

BRESLOW, N. (1970). A generalised Kruskal-Wallis test for comparing k samples subject to unequal patterns of censorships. *Biometrika*, **57,** 579.

CANO, A., COFFER, A.I., ADATIA, R., MILLIS, R.R., RUBENS, R.D. & KING, R.J.B (1986). Histochemical studies with an oestrogen receptor related protein in human breast tumours. *Cancer Res.*, **46,** 6475.

COFFER, A.I., MILTON, P.T., PRIZE-DAVIES, J. & KING, R.J. (1977). Purification of oestradial receptor from human uterus by affinity chromatography. *Cell Endocrinol*, **6,** 231.

DIXON, W.J. (1985). (ed.) *BMDP Biomedical Computer Programs.* Berkeley: University of California Press.

ESTIVAL, A., MOUNIELOU, P., TROCHERIS, V. & 4 others (1983). Presence of VIP receptors in a human pancreatic adenocarcinoma cell line. Modulation of the CAMP response during cell proliferation. *Biochem. Biophys. Res. Com.*, **111,** 958.

HARRISON, J.D., MORRIS, D.L., ELLIS, I.O., JONES, J. & JACKSON, I. (1989). The effect of tamoxifen and oestrogen receptor status on survival in gastric carcinoma. *Cancer*, **64,** 1007.

HEYDERMAN, E., EBBS, S.R., LARKIN, S.E., BOUN, B.M.E., HAINES, A.M.R. & BATES, T. (1989). Response of breast carcinoma to endocrine therapy predicted using immunostained pelleted fine needle aspirates. *Br. J. Cancer*, **60,** 630.

KING, R.J.B., COFFER, A.I., GILBERT, J. & 5 others (1985). Histochemical studies with a monoclaon antibody raised against a partially purified soluble estradiol receptor preparation from human myometrium. *Cancer Res.*, **45,** 5728.

LEAKE, R.E., LAING, C., CALMAN, K.C. & MACBETH, F.R. (1980). Estrogen receptors and antiestrogen therapy in selected human solid tumours. *Cancer Treat Rep.*, **64,** 797.

MANTEL, N. (1966). Evaluation of survival data and two new rank order statistics arising in its considerating. *Cancer Chemotherapy Rep.*, **50,** 163.

MCCLEADON, J.E., APPLEBY, D., CLAUDON, D.B., DONEGAN, W.L. & DECROSSE, J.J. (1977). Colonic neoplasms – tissue oestrogen receptor and carcinoembryonic antigen. *Arch. Surg.*, **112,** 240.

NISKI, K. & ? others (1987). Immunohistological study of intracellular oestradiol in human gastric cancer. *Cancer*, **59,** 1328.

RAE-VENTER, B., TOWNSEND, C.M., THOMPSON, J.C. & SINAN, D.M. (1981). Gastrin receptors in cultured human cells derived from carcinoma of the colon, stomach and pancreas. *Endocrinology*, **1108** (Suppl): A153.

SICA, V., NOLA, E., CONTIERE, & 7 others (1984). Estradiol and progesterone receptors in malignant gastrointestinal tumours. *Cancer Res.*, **44,** 4670.

VIGUERIE, N., TAHIRI JOUTI, N. & 6 others (1980). Functional somatostatin receptors on a rat pancreatic acinar cell line. *Am. J. Physiol.*, **255,** G113.